# Management of adnexal masses’ torsion during pregnancy

**DOI:** 10.11604/pamj.2020.37.17.23869

**Published:** 2020-09-04

**Authors:** Alpha Boubacar Conte, Solène Nyingone, Sofia Jayi, Bineta Jho Diagne, Fatima Zohra Fdili Alaoui, Hikmat Chaara, Moulay Abdelilah Melhouf

**Affiliations:** 1 Sidi Mohamed Ben Abdellah University, Department of Gynecology and Obstetrics II, Hassan II Teaching Hospital, Fez, Morocco,; 2 Sidi Mohamed Ben Abdellah University, Laboratory of Epidemiology, Clinical Research and Community Health, Fez, Morocco

**Keywords:** Torsion, adnexal mass, pregnancy, management

## Abstract

With the increased use and quality of ultrasound in pregnancy, adnexal masses are being encountered with greater frequency. Most of the time such masses are asymptomatic. It can be discovered in an emergency. Surgical intervention may cause risks to the mother and her fetus, while observation without intervention may also lead to unfavorable complications, such as ovarian torsion or the development of a tumor. Therefore, the management requires a balance between the maternal and fetal risks. We report two cases of torsion of adnexal masses during pregnancy, and we provide a brief literature review on the management and prognosis of this condition in pregnancy.

## Introduction

With the increased use and quality of ultrasound in pregnancy, adnexal masses are being encountered with greater frequency. Fortunately, the vast majority of such masses is benign and resolve on their own. However, it is important for clinicians to be familiar with the types of adnexal masses that may be visualized in pregnancy to best counsel these women. In addition, complications such as ovarian torsion, and rarely, even malignancy can occur [1]. This situation is often a source of diagnostic problems, which could lead wrongly to surgical management [2]. Conservative treatment is discussed outside the emergency; however, the occurrence of acute complications such as torsion of the appendix requires more or less invasive surgical procedures [3]. Surgical intervention may cause risks to the mother and her fetus, while observation without intervention may also lead to unfavorable complications, such as ovarian torsion or the development of a tumor [4].

## Patient and observation

**Figure 1:**
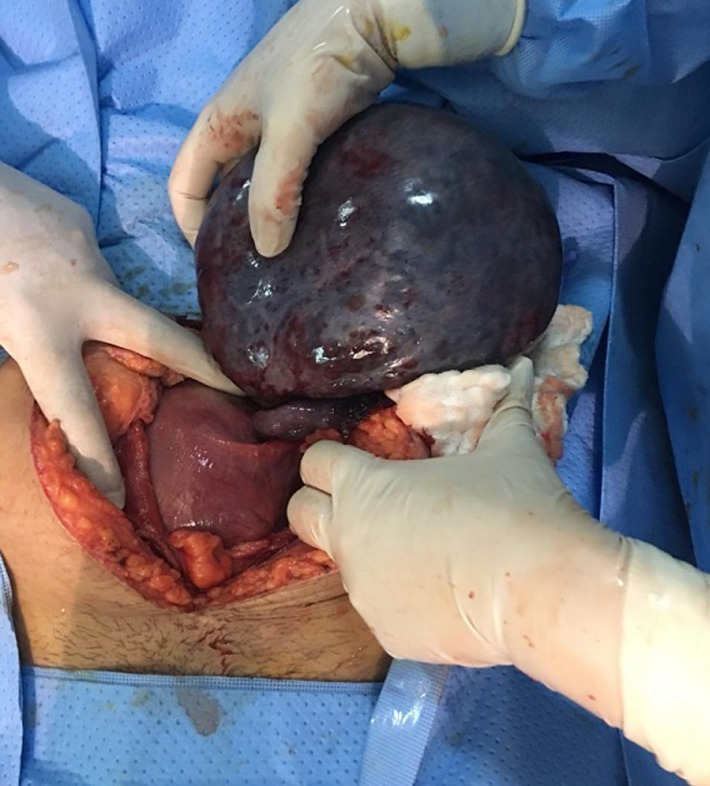
cyst torsion of the left ovary

**Case 1:** a 39-year-old patient, G2P0 known to have an ovarian cyst who consulted for acute pelvic pain in a pregnancy of 18 weeks and 6 days in which the clinical examination found a stable and non-pyretic patient with generalized abdominal defense. On gynecological examination: the cervix was macroscopically normal, no bleeding from the endocervix, the uterus was enlarged with the presence of a left lateral uterine pain. Obstetrical ultrasound found a progressive pregnancy with a gestational age estimated at 18 weeks. Presence at the left uterine side of an anechoic image with a thin wall, without endo or exocystic vegetation and without partition at the expense of the left ovary measuring 15/12cm in favor of an ovarian cyst. In front of the severe pain and the existence of an ovarian cyst the diagnosis of ovarian torsion was retained and the patient underwent a laparotomy. The procedure was done under rachi anesthesia. The exploration found a large cyst of the left ovary about 15cm with a thin wall without vegetation with several turns of the coil and necrosis of the left appendix (Figure 1). We realized a distortion of the ovary which remained necrotic. Thus we performed a left adnexectomy. The pregnancy was carried until the end with a successful vaginal delivery.

**Case 2:** an 18-year-old patient who consulted for a syncopal acute pelvic pain in whom clinical examination found a generalized abdominal defense more marked in the right side making the rest of the exam difficult. Obstetrical ultrasound found a uterus increased of size with a progressive pregnancy with a fetus´s crown rump length corresponding to 14 weeks + 2 days and presence at the right side of a para tubal cystic image of 8cm not taking the doppler with a medium abundance effusion. The patient underwent emergency laparotomy and the exploration found the presence of a medium-abundance effusion and the presence at the right adnexa of a large mass of 10cm performing 6 turns of coil and a necrosis of the right adnexa. The left side adnexa was normal. The right adnexectomy was done. The pregnancy was carried until the end with a successful vaginal delivery.

## Discussion

The incidence of adnexal masses in pregnancy has increased tremendously with the routine application of ultrasound for pregnancy surveillance. It varied from 1/76 to 1/2328 deliveries in a recent study [5]. The majority of adnexal masses are discovered incidentally during routine prenatal ultrasound performed for obstetric indications [6]. These account for about 30% of masses in pregnancy and usually regress spontaneously during the first or early second trimester of gestation [7]. Clinically, there´s no specific symptomatology of adnexal mass during pregnancy. Most women do not refer any symptoms. When symptomatic, the most common symptom described is abdominal pain [8]. The clinical exam might be poor and not relevant at the second or third trimester of pregnancy due to the enlargement of the uterus and the change in the anatomical situation of adnexa. According to D´Ambrosio *et al*. [7], if a pelvic mass is identified, a complete physical examination is required and the first step is to exclude bladder dysfunction with retained urine. It is also mandatory to exclude infectious diseases or extra ovarian cancer. In case of extra ovarian cancer, it is necessary to exclude cervical/supraclavicular/groin lymphadenopathy, pleural effusion, or ascites. Breast examination is also mandatory because the ovary can be a metastasis site for breast cancer. The main features of the adnexal mass (size, location, consistency, and mobility) can be evaluated with rectovaginal and bimanual pelvic examination [7].

Ultrasound is the preferred initial imaging study to assess an adnexal mass, both in and out of pregnancy [6]. It has specifically been shown to be accurate in characterization of adnexal masses during pregnancy [3]. Various mass characteristics can be evaluated with an ultrasound examination, for example, definition of diameters, morphology, vascularization and growth, evaluation of contralateral adnexa and identification of other suspicious signs for neoplasm such as ascites and peritoneal carcinomatosis [8]. The International Ovarian Tumour Analysis (IOTA) classification has been used to characterize adnexal masses. It has been suggested that neoplasms are irregular solid tumor; multilocular and irregular masses of 10cm or more; presence of septa with a thickness of 2-3 mm; presence of at least 3 papillary projections; increased vascularization; evidence of ascites; or peritoneal masses. Even though there are no data showing the efficacy of this model in pregnancy, it is probable that its validity is applicable also in evaluating masses during pregnancy [8,9]. In case of non-conclusive ultrasound, or when a wider assessment of tissue planes and relation to other organs may be important in both obstetric and surgical planning, magnetic resonance imaging (MRI) can be a useful. Non-contrast MRI has been safely used in pregnancy and does not appear to be harmful to the mother or the fetus. Advantages include a larger scanning and improved definition of tissue planes and their composition [6].

The management of adnexal masses in pregnancy is controversial. It might be a threat for the pregnancy. Surgical intervention may cause risks to the mother and her fetus, while observation without intervention may also lead to unfavorable complications, such as ovarian torsion or the development of a tumor [4]. The management requires a balance between the maternal-fetal risks of surgery, the risks of mass-related complications, and the likelihood of a malignancy, all of which are considered within each patient´s distinct clinical scenario and gestational age [6]. Emergency surgery should be done as soon as possible, for patients with acute abdominal pain with a high suspicion of ovarian torsion [3]. For patients without symptoms, it´s suggested that observation is adequate since most adnexal masses are functional ovarian cysts and will spontaneously resolve [3,10]. When the surgery takes place between 24 to 34 weeks´ gestation, a prophylactic course of antenatal corticosteroids may be considered for fetal lung maturity given a possible increase in risk for preterm delivery during this gestational age window [6].

## Conclusion

Discovering adnexal mass during pregnancy is not common situation even though it has been increased by the systematic use of ultrasound for the pregnancy surveillance. The management is described to be controversial, as some masses do not need to be managed. The decision of surgery should be taken after the assessment of the balance between the maternal and fetal risks.
